# The genome of *Draba nivalis* shows signatures of adaptation to the extreme environmental stresses of the Arctic

**DOI:** 10.1111/1755-0998.13280

**Published:** 2020-11-12

**Authors:** Michael D. Nowak, Siri Birkeland, Terezie Mandáková, Rimjhim Roy Choudhury, Xinyi Guo, Anna Lovisa S. Gustafsson, Abel Gizaw, Audun Schrøder‐Nielsen, Marco Fracassetti, Anne K. Brysting, Loren Rieseberg, Tanja Slotte, Christian Parisod, Martin A. Lysak, Christian Brochmann

**Affiliations:** ^1^ Natural History Museum University of Oslo Oslo Norway; ^2^ CEITEC Masaryk University Brno Czech Republic; ^3^ Institute of Plant Sciences University of Bern Bern Switzerland; ^4^ Science for Life Laboratory and Department of Ecology Environment and Plant Science Stockholm University Stockholm Sweden; ^5^ Centre for Ecological and Evolutionary Synthesis Department of Biosciences University of Oslo Oslo Norway; ^6^ Department of Botany The University of British Columbia Vancouver BC Canada

**Keywords:** adaptation, Arctic, Brassicaceae, chromosome‐scale assembly, linkage map

## Abstract

The Arctic is one of the most extreme terrestrial environments on the planet. Here, we present the first chromosome‐scale genome assembly of a plant adapted to the high Arctic, *Draba nivalis* (Brassicaceae), an attractive model species for studying plant adaptation to the stresses imposed by this harsh environment. We used an iterative scaffolding strategy with data from short‐reads, single‐molecule long reads, proximity ligation data, and a genetic map to produce a 302 Mb assembly that is highly contiguous with 91.6% assembled into eight chromosomes (the base chromosome number). To identify candidate genes and gene families that may have facilitated adaptation to Arctic environmental stresses, we performed comparative genomic analyses with nine non‐Arctic Brassicaceae species. We show that the *D. nivalis* genome contains expanded suites of genes associated with drought and cold stress (e.g., related to the maintenance of oxidation‐reduction homeostasis, meiosis, and signaling pathways). The expansions of gene families associated with these functions appear to be driven in part by the activity of transposable elements. Tests of positive selection identify suites of candidate genes associated with meiosis and photoperiodism, as well as cold, drought, and oxidative stress responses. Our results reveal a multifaceted landscape of stress adaptation in the *D. nivalis* genome, offering avenues for the continued development of this species as an Arctic model plant.

## Abstract 2

see also the Perspective by Tanja Pyhäjärvi and Tiina M. Mattila

## 1 INTRODUCTION

The Arctic accounts for ~10% of Earth's land surface, and the combination of high latitude and regional climate patterns make it one of the harshest terrestrial environments on the planet. Arctic plants must endure an extremely short and unpredictable growing season, with mean July temperatures ≤ 10°C and up to 24 hr of sunlight. The climatic conditions impose strong selective pressures for managing cellular and physiological responses to environmental stresses, including cold, drought (Lütz, 2010), and high light exposure (Caldwell et al., 2007). Little is known about the molecular basis of Arctic plant adaptations, and an Arctic model species has yet to be developed to facilitate such studies (Colella et al., 2020; Wullschleger et al., 2015). Exploring the molecular basis of Arctic plant adaptation may reveal novel mechanisms of environmental stress tolerance, potentially offering guidance to agricultural crop improvement.

Here, we present the chromosome‐scale genome assembly of *Draba nivalis*, a perennial diploid with a circum‐Arctic distribution. This species is ideal for studying the evolution of environmental stress tolerance in plants because it occurs in the high Arctic where extremes in temperature, light regime, and low water availability are ever‐present. In a recent study of its transcriptome, we identified numerous candidate genes for Arctic adaptation illuminating its potential as an Arctic model species (Birkeland et al., 2020). *Draba nivalis* is also an emerging model for studying the genetics of incipient speciation as intraspecific crosses frequently result in highly sterile hybrids (Grundt et al., 2006; Gustafsson et al., 2014; Skrede et al., 2008). *Draba* is the largest genus in the Brassicaceae with > 390 species, which mainly occur in Arctic and alpine regions (Jordon‐Thaden et al., 2013). Many of these species, including *D. nivalis*, form dense and hairy cushions as protection from wind and cold, and are strongly autogamous to secure reproduction in their pollinator‐poor environments (Brochmann, 1993). The Brassicaceae contains numerous species important for agriculture as well as for research in plant ecology, evolution, development, and molecular biology (Gupta, 2016). The availability of many Brassicaceae genome assemblies enabled us to conduct comparative analyses of chromosomal evolution and functional genomics, to shed light on the genomic characteristics of a plant adapted to the extreme abiotic stresses of the Arctic.

## 2 MATERIALS AND METHODS

### 2.1 Plant material and DNA sequencing

Seeds of *D. nivalis* accession 008‐7 from Alaska (Waterfall Creek W., 63.045 latitude, –147.201 longitude; (see Grundt et al., 2006 for complete locality information) were grown under conditions mimicking the Arctic climate (specified in Brochmann et al., 1992) in the Phytotron at the Department of Biosciences, University of Oslo. Genomic DNA was extracted from young leaf tissue using the Qiagen Plant Mini Kit, and 7.7 µg of DNA was delivered to the Norwegian Sequencing Centre for library preparation and sequencing. Paired‐end sequencing libraries with a target insert size of 550 bp were produced using the Illumina TruSeq PCR‐free kit. This library was sequenced on one half of each of two Illumina HiSeq 2,500 flow cells in rapid run mode with a read length of 250 bp. These data were used to estimate the genome size based on 25 mer frequency using GenomeScope (Vurture et al., 2017; http://qb.cshl.edu/genomescope/). A total of 8 µg of genomic DNA was used to produce four sequencing libraries for the Oxford Nanopore MinION platform. For each library, 2 µg of genomic DNA was treated with NEBNext Ultra II End‐Repair/ dA‐tailing Module and libraries were prepared using the Oxford Nanopore 1D ligation kit (SQK‐LSK108) following the manufacturer's protocol. Each of these four libraries was sequenced on a single Oxford Nanopore MinION flow cell (version R9) for approximately 48 hr. Two whole plants of *D. nivalis* accession 008‐7 were flash frozen in liquid nitrogen and shipped to Dovetail Genomics (LLC, Santa Cruz, California 95060, USA) on dry ice for Chicago proximity ligation library preparation and 150 bp paired‐end sequencing on the Illumina HiSeq 2500 platform.

### 2.2 Genome assembly and scaffolding

The first draft assembly of *D. nivalis* accession 008‐7 was produced using all (approximately 270 million 250 bp paired‐end reads) unfiltered Illumina HiSeq data with the software DISCOVAR de novo (release 52488; https://software.broadinstitute.org/software/discovar/blog/) using default parameter settings. This draft assembly (scaffold N50 = 30.083 Kb) was supplied to Dovetail Genomics for scaffolding with the HiRise pipeline using 150 bp Chicago reconstituted chromatin paired‐end reads. The resulting scaffolded draft assembly (scaffold N50 = 2.92 Mb) was further improved by scaffolding with approximately 796 Mb of Oxford Nanopore 1D long reads passing the default quality filtration score in the Metrichor base calling pipeline. Long read scaffolding was first conducted using SSPACE‐LongRead version 1.1 (Boetzer & Pirovano, 2014) using a minimum alignment identity of 90. These scaffolds were further improved with the Oxford Nanopore long reads using links version 1.8.6 (Warren et al., 2015) with K‐mer size set to 21 and using 17 different distances between K‐mer pairs (i.e., –d 1 Kb, 2 Kb, 4 Kb, 6 Kb, 7 Kb, 8 Kb, 10 Kb, 12 Kb, 14 Kb, 16 Kb, 18 Kb, 21 Kb, 25 Kb, 30 Kb, 40 Kb, 50 Kb, 60 Kb).

To compare broad patterns of synteny, the *D. nivalis* genome was aligned to the genomes of *A. alpina* (the most closely related species with an assembled genome; Guo et al., 2017) and *A. lyrata* with NUCmer version 4.0b2 (Kurtz et al., 2004) using all anchor matches regardless of their uniqueness (‐‐maxmatch) and setting the minimum length of a cluster of matches (‐c) to 100.

### 2.3 Genetic map construction and final map‐based scaffolding

We generated an F_2_ mapping population by self‐pollination of an F_1_ hybrid obtained from a cross between a *D. nivalis* plant from Norway (045‐5; maternal parent; see Grundt et al., 2006 for complete locality information) and a *D. nivalis* plant from Alaska (008‐7; paternal parent). Seeds were gently scarified before sowing and a total of 575 F_2_ individuals were grown to maturity under our phytotron conditions (see above). Genomic DNA was extracted from young leaf tissue using the Qiagen Plant Mini Kit, and 96‐plex double‐digest restriction‐associated DNA (ddRAD) libraries were produced. For the ddRAD procedure, the restriction enzymes *NsiI* and *MseI* were used to digest 500 ng of genomic DNA per sample. Indexed P1 and P2 adapters with sticky ends matching the overhangs left by the restriction enzymes were added to the digested DNA. Following adapter ligation, individual indexed libraries were pooled and amplified with an eight cycle PCR. Ampure XP bead cleanup was performed to remove short fragments (i.e., less than ~200 bp), and the multiplexed libraries were visualized on an Advanced Analytical Fragment Analyser to ensure the libraries were of the correct size (i.e., 300–450 bp). See Supporting Information Methods for protocol details. The final multiplexed libraries were sequenced on six Illumina HiSeq 2500 lanes by the Norwegian Sequencing Centre.

Reads were demultiplexed using ipyrad version 0.5.15 (Eaton, 2014), and adapters and low‐quality reads were removed using Cutadapt and FastQC available in the wrapper script Trim Galore! version 0.4.5 (http://www.bioinformatics.babraham.ac.uk/projects/trim_galore/; bases with a Phred score less than 20 were trimmed, and reads shorter than 35 bp following trimming were discarded). A total of six F_2_ individuals were removed because of low quality reads. Trimmed paired‐end sequence reads were each mapped to the Dniv0087_Chicago assembly using bwa‐mem version 0.7.8 (Li, 2013; Li & Durbin, 2010) with default settings, and duplicate reads were filtered using MarkDuplicates in Picard version 2.0.1 (http://broadinstitute.github.io/picard/). BAM alignment processing and SNP calling were performed with the Genome Analysis Toolkit version 4.beta6 (gatk; McKenna et al., 2010). Briefly, GATK RealignerTargetCreator and IndelRealigner were first used to realign indels, and base quality scores were recalibrated using GATK BaseRecalibrator and PrintReads with default settings. Indels and SNPs were called using gatk UnifiedGenotyper in DISCOVERY mode using default parameters. Indels were discarded with VCFtools version 0.1.13 (Danecek et al., 2011) resulting in a VCF file with a total of 166,644 SNPs prior to filtration. VCFtools version 0.1.13 was used to isolate biallelic SNPs and exclude any SNPs that mapped to regions of the Dniv0087_Chicago genome assembly annotated as repetitive elements (see below for repetitive element annotation). VCFtools was further used to filter the biallelic SNPs for sites with a minimum and maximum coverage depth of eight and 200, respectively, sites with a minimum mapping quality of 50, sites with a minor allele frequency greater than 0.0001, and sites that were called in at least 95% of the samples. These filtration steps resulted in a final VCF file containing 13,990 SNPs genotyped in 537 F_2_ individuals.

Using these data, we constructed a genetic map using R/qtl version 1.42‐8 (Arends et al., 2010; Broman et al., 2003) and ASMap version 1.0‐4 (Taylor & Butler, 2017). Individuals with more than 5% missing genotypes and those that represented duplicate genotypes were removed. Loci that represented redundant genotypes were removed and an initial genetic map was estimated. Based on this map, 2,086 markers exhibiting significant segregation distortion (*p*‐value < 1e‐10) were removed and map distances were recalculated. The cross upon which this map is based is expected to contain biological sources of segregation distortion as hybrid progeny of these genotypes have previously been shown to exhibit both seed and pollen infertility (Grundt et al., 2006; Gustafsson et al., 2014; Skrede et al., 2008). To ensure that we had a genetic map as complete as possible upon which to scaffold the genome assembly, we reintegrated distorted markers into the genetic map using ASMap. The resulting genetic map and the first map that contained the distorted markers were imported into ASMap and markers exhibiting segregation distortion at ‐log_10_ *p*‐value < 6 were then pushed back into the map based on the marker order reflected by the initial map, and map distances were estimated again without changing the marker ordering. The final genetic map contained 5,055 markers genotyped in 480 F_2_ individuals (Figure S1, Table S1).

Chromonomer version 1.08 (http://catchenlab.life.illinois.edu/chromonomer/) was used with default settings to scaffold the assembly based on the genetic map. To create the input files necessary to run Chromonomer, the ddRAD loci containing SNPs constituting the genetic map were first aligned to the scaffolded *D. nivalis* accession 008‐7 draft genome assembly using bwa‐mem (Li, 2013; Li & Durbin, 2010), and an AGP file was then generated for the scaffolded genome assembly using the script fatoagp.pl (https://github.com/sjackman/fastascripts/blob/master/fatoagp).

### 2.4 Identification and annotation of transposable elements

LTR‐RTs were annotated following Choudhury et al. (2017) by identifying full‐length LTR‐RT copies based on structural features using ltrharvest 1.5 (Ellinghaus et al., 2008). After removal of nested as well as overlapping elements, candidate copies with internal regions matching plant non‐LTR retrotransposon or DNA transposon consensus sequences from Repbase (www.girinst.org/repbase/; accessed in June 2018; Ellinghaus et al., 2008) were excluded. Internal coding regions and binding sites of remaining candidate full‐length copies were annotated and classified into *Gypsy* and *Copia* superfamilies using ltrdigest (Steinbiss et al., 2009), using Hidden Markov models based on plant LTR‐RT protein (Gag, Reverse transcriptase, Protease, RNaseH, Integrase, Chromodomain, and Envelope; downloaded from www.gydb.org; Llorens et al., 2011) and eukaryotic tRNA entries from the UCSC gtRNA database. Terminal inverted repeat transposons (TIR) were identified based on the presence of TIR sequences flanked by target site duplications, using GenomeTools tirvish (Gremme et al., 2013). After removal of sequences nested with other known transposable elements, putative full‐length copies of TIR transposons were confirmed by inspecting internal coding domains of Class II transposons using Hidden Markov models. Helitron sequences were identified with HelitronScanner (Xiong et al., 2014) which searches for upstream and downstream termini of Helitrons within 200–20,000 bp of each other, in either the direct and reverse complement orientation following Dunning et al. (2019).

Copies of transposable elements identified in *D. nivalis* were classified into families by clustering sequences with at least 80% similarity (i.e., technical definition; El‐Baidouri & Panaud, 2013; Wicker et al., 2009) using CD‐HIT‐EST (Li & Godzik, 2006). For LTR‐RTs, clustering was based on LTR sequences. The full‐length copy showing the lowest E‐value to an HMM profile in each cluster was selected for the classification of family based on longest significant blastn hits of reverse‐transcriptase domains flanked by 800 bp on either side against corresponding reverse‐transcriptase sequences from Brassicaceae. Classified Brassicaceae families were further assigned to “tribes” following Choudhury et al. (2017). For TIR transposons, clustering was based on full‐length copies for TIR transposons that were further classified into superfamilies (Harbinger, hAT, Mariner/Tc1, MuDR, EnSpm/CACTA) based on longest significant blastn hits against Viridiplantae DNA transposons extracted from Repbase. Helitrons show notoriously diverse internal regions and were classified following Yang and Bennetzen (2009), with clustering based on identity over 30 bp at the 3’ end of copies (i.e., the hairpin‐forming region crucial for rolling circle replication).

Transposable elements were annotated along the genome assembly using all structurally defined and hierarchically classified copies of LTR‐RTs, TIR transposons and helitrons from *D. nivalis* together with non‐LTR‐RTs (i.e., LINE and SINE from Viridiplantae in Repbase) as a reference. After removal of sequences giving significant blast hit with swissprot protein database for plants and with other transposable element sequences, this reference was used in RepeatMasker (version Open‐4; http://www.repeatmasker.org) with RM‐BLAST as search engine and divergence set to 20%. Resulting annotations of remnants of transposable element sequences were filtered to remove nested copies and copies with less than 80 bp.

### 2.5 Transcriptome assembly and gene annotation

Four tissues were sampled from *D. nivalis* accession 008‐7 for RNA extraction: young leaves (approximately 2–7 days following leaf blade expansion), mature floral buds (approximately 2–4 days prior to anthesis), open flowers (approximately 1–3 days post‐anthesis), and root tissues (thoroughly washed of soil). Total RNA was isolated using the Thermo Fisher RNAqueous‐Micro Kit following the manufacturers standard protocol for fresh tissues. A total of 30.6 µg (leaves), 22 µg (flower buds), 23 µg (open flowers), and 3 µg (roots) of total RNA was provided to the Norwegian Sequencing Centre for Illumina TruSeq Stranded RNA library preparation and sequencing. Each library was sequenced on 1/10th of an Illumina HiSeq 2500 lane to generate 125 bp paired‐end reads. A de novo transcriptome assembly was generated for *D. nivalis* accession 008‐7 using the RNA‐seq data from all four tissues with the Trinity version 2.4.0 pipeline (Grabherr et al., 2011; Haas et al., 2013) including default read quality trimming and filtration using Trimmomatic version 0.32 (Bolger et al., 2014).

Genes were predicted in the *D. nivalis* genome assembly using the Maker version 2.31.9 (Holt & Yandell, 2011) pipeline. Augustus version 3.2.2 (Stanke et al., 2008) and SNAP (Release 2013‐11‐29; Korf, 2004) were used as ab initio gene predictors. The Trinity transcriptome assembly (see above) was used as transcript evidence, and protein sequences from the species *Arabis nemorensis, Eutrema salsugineum, Arabis alpina, Arabidopsis lyrata,* and *Arabidopsis thaliana* (version TAIR10) were used as homology‐based evidence. The *D. nivalis* repeat library (see above) was included to mask repetitive elements from annotation. The Maker annotation was first run using the *D. nivalis* transcriptome directly to infer gene predictions, and training files for the ab initio gene predictors were produced with these results. Maker was run iteratively three additional times using the transcriptome as evidence and providing updated training files for each run. The resulting set of predicted genes was annotated with Pfam domains (El‐Gebali et al., 2018) using InterProScan verion 5.4–47.0 (Jones et al., 2014), and GO terms were annotated using Blast2GO version 5.2.5 (Conesa et al., 2005) by searching against the UniProt (https://www.uniprot.org/) database for Viridiplantae. Both the *D. nivalis* genome assembly and the predicted gene set were also evaluated for completeness by searching against a set of 1,440 highly conserved plant genes (Embryophyta) using busco version 3.0.1 (Simao et al., 2015). The genome assembly and predicted gene set were assessed for completeness by running BUSCO in both “genome” and “prot” modes, respectively.

### 2.6 Comparative chromosome painting

Whole inflorescences of *D. nivalis* were fixed in freshly prepared ethanol:acetic acid fixative (3:1) overnight, transferred into 70% ethanol and stored at –20°C until use. Mitotic and meiotic (pachytene and diakinesis) chromosome preparations were prepared as described by Mandáková and Lysak (2016a) on suitable slides pretreated with RNase (100 µg/ml, AppliChem) and pepsin (0.1 mg/ml, Sigma‐Aldrich). Based on the known chromosome structure of *A. alpina* and other Arabideae species (Mandáková et al., 2020; Willing et al., 2015), representative BAC clones of *A. thaliana* were selected and grouped into contigs for comparative chromosome painting (CCP). A total of 5–10 BAC clones from each tested genomic region of *A. alpina* were used as hybridization probes on mitotic chromosome spreads in *D. nivalis* (Figure S2 Dniv1: genomic regions A and B; Dniv2: D and E; Dniv3: Fa and Fb; Dniv4: C, T and Jb; Dniv5: K‐L, M‐Na and M‐Nb; Dniv6: O, V and S; Dniv7: Ua and Ub; Dniv8: R, W and X). The four most reshuffled chromosomes of *A. alpina* and other Arabideae species (Mandáková et al., 2020; Willing et al., 2015) were investigated in detail by hybridization of whole‐chromosome paints (i.e. BAC contigs covering whole chromosomes except pericentromere) on pachytene chromosomes of *D. nivalis* (Figure 3; Dniv4, Dniv5, Dniv6 and Dniv7). The *A. thaliana* BAC clone T15P10 (AF167571) bearing 35S rRNA gene repeats was used for in situ localization of nucleolar organizer regions, and the clone pCT4.2 (M65137), corresponding to a 500 bp 5S rDNA repeat, was used to localize 5S rDNA loci (Figure S3). All BACs and rDNA probes were labelled with biotin‐dUTP, digoxigenin‐dUTP, or Cy3‐dUTP by nick translation as described by Mandáková and Lysak (2016b). The labelled BACs were pooled together, ethanol precipitated, dissolved in 20 µl of hybridization mixture (50% formamide and 10% dextran sulphate in 2 × SSC) per slide and pipetted to a microscopic slide containing chromosome spreads. The slide was heated at 80°C for 2 min and incubated in a moist chamber at 37°C overnight. Hybridized probes were visualized either as the direct fluorescence of Cy3‐dUTP or through fluorescently labelled antibodies against biotin‐dUTP and digoxigenin‐dUTP following Mandáková and Lysak (2016b). Chromosomes were counterstained with 4,6‐diamidino‐2‐phenylindole (DAPI, 2 µg/ml) in Vectashield antifade. Fluorescent signals were analysed and photographed using a Zeiss Axioimager epifluorescence microscope and a CoolCube camera (MetaSystems). Individual images were merged and processed using Photoshop CS6 software (Adobe Systems).

### 2.7 Analyses of gene family evolution

To compare the *D. nivalis* genome assembly with other Brassicaceae species whose genomes have been sequenced, whole genome assemblies and associated gene annotations were downloaded from public databases (Table S2) for the following nine species (representing three Brassicaceae clades, Guo et al., 2017): *Arabis alpina* (clade B), *Arabidopsis lyrata* (clade A), *Arabidopsis thaliana* (clade A), *Capsella rubella* (clade A), *Raphanus raphanistrum* (clade B), *Brassica oleracea* (clade B), *Thellungiella parvula* (clade B), *Thlaspi arvense* (clade B), and *Aethionema arabicum* (clade F). Among these species, *A. alpina* is thought to be the most closely related to *D. nivalis* (clade B) with an assembled genome (Guo et al., 2017). As a first analysis of gene content, we annotated Pfam domains (El‐Gebali et al., 2018) for the predicted genes of each assembly using InterProScan (Jones et al., 2014). Pfam domains were quantified for each species, and domains with a Z‐score above 1.96 or below –1.96 in *D. nivalis* were considered significantly enriched or contracted, respectively.

To estimate gene family composition and membership Orthofinder version 2.2.7 (Emms & Kelly, 2015, 2019) was run using the proteins annotated in the *D. nivalis* genome and the nine Brassicaceae genomes. OrthoFinder was run with default settings using MMseqs2 (Steinegger & Söding, 2017) to cluster proteins by sequence similarity. Tests for significant contractions and expansions of gene families (defined as “orthogroups” by OrthoFinder) were performed with cafe version 4.2 (Han et al., 2013). The species tree used for the CAFE analysis was generated by OrthoFinder using stag (Emms & Kelly, 2018) and rooted using stride (Emms & Kelly, 2017). This species tree was transformed into an ultrametric tree using r8s (Sanderson, 2003) by fixing the age of the most recent common ancestor of *A. arabaicum* and the remaining nine Brassicaceae species to 35.2 Ma based on the divergence times reported by Guo et al. (2017). We classified gene duplications within the *D. nivalis* genome using the Dup_GenFinder pipeline (Qiao et al., 2019). This pipeline used the results of an all‐versus.‐all BLASTp of the *D. nivalis* gene set to itself, and BLASTp results comparing the *D. nivalis* gene set to a closely related species (here the *A. alpina* v.4 genome assembly was used; Willing et al., 2015) to identify homologous gene pairs. mcscanx (Wang et al., 2012) was used to identify patterns of synteny and collinearity in the duplicated genes, both within the *D. nivalis* genome and between *D. nivalis* and *A. alpina*. Dup_GenFinder synthesizes the outputs of these analyses to classify gene duplications into one of five categories using methods that are described in detail in Qiao et al. (2019) and Wang et al. (2012). Tandem duplications (TD) are gene pairs that are located next to one another on the same *D. nivalis* chromosome, probably resulting from unequal crossing over. Proximal duplications (PD) represent gene pairs that are located on the same *D. nivalis* chromosome and separated from one another by 10 or fewer genes, probably resulting from local transposon activity or ancient TD events. Transposed duplications (TRD) are gene pairs comprised of ancestral and novel copies, which are defined based on intra‐ and interspecies collinearity (Qiao et al., 2019), and are probably the product of distant RNA or DNA transposon activity. Whole genome duplication (WGD) is inferred for genes that reside in relatively large collinear chromosomal regions (collinear blocks) shared by *D. nivalis* and *A. alpina* (also called segmental duplicates by Wang et al., 2012). Dispersed duplications (DSD) are gene pairs that do not fulfill the criteria for classification into one of the previous four categories, and while the mechanisms responsible for their proliferation remain unclear, such distant single gene translocations may be mediated by several types of DNA transposons (Wang et al., 2012).

### 2.8 Positive selection tests

We used the branch‐site model (Zhang et al., 2005) implemented in codeml of paml version 4.9i (Yang, 1997) to test for site‐wise positive selection happening on the branch leading to *D. nivalis*. Briefly, *D. nivalis* was defined as the foreground branch on a predefined phylogeny consisting of eight Brassicaceae species (*T. parvula*, *B. oleraceae*, *R. raphanistrum*, *A. alpina*, *C. rubella*, *A. thaliana*, *A. lyrata*, and *D. nivalis*; Table S2), and two models were compared with a likelihood ratio test (LRT): an alternative model that allowed positive selection on the foreground branch, and a null model that did not allow positive selection on the foreground branch (omega fixed to 1). The alternative model was accepted if *p* < .05 (using *χ* ^2^ with one degree of freedom), implying that positive selection has acted on a subset of sites along the branch leading to *D. nivalis*. The test was run on orthologous gene‐alignments with one gene copy from all eight species, constructed from orthogroups identified with OrthoFinder (orthogroups are genes descended from a single gene in the last common ancestor of the eight species; Emms & Kelly, 2015, 2019). Due to the low number of single‐copy orthogroups, multiple copy orthogroups were divided into subsets based on the smallest genetic distance to each of the *D. nivalis* gene copies. This was achieved by (a) aligning all orthogroups based on protein sequence using mafft (Katoh et al., 2005); (b) calculating Kimura protein distances (Kimura, 1983) with the distmat algorithm in emboss version 6.6.0 (Rice et al., 2000); and (c) extracting one gene copy from all Brassicaceae species based on the smallest protein distance to each *D. nivalis* gene copy. The resulting orthogroup subsets were realigned using prank (Löytynoja & Goldman, 2005) in guidance version 2.02 (Sela et al., 2015) with 10 bootstraps. guidance enables identification and filtration of unreliable alignment regions and sequences, and has been shown to improve positive selection inference on simulated data when used in combination with a phylogeny aware aligner like prank (Jordan & Goldman, 2012; Privman et al., 2012). All alignments containing sequences scoring < 0.6 and all alignment columns scoring < 0.8 in guidance were removed from the data set. Codeml was run 3–4 times for each model with different initial parameter values, and the run with the highest likelihood score was used in the final LRT (see e.g., Wong et al., 2004). Sites with ambiguity data were removed within codeml, and the species phylogeny inferred in OrthoFinder was used in all runs.

### 2.9 Gene ontology enrichment tests

The positively selected gene set and the sets of expanded and contracted gene families were tested for overrepresented gene ontology (GO) terms using the Bioconductor package topGO version 2.34 (Alexa et al., 2006; Gentleman et al., 2004). We used a Fisher's exact test in combination with the “classic”, “elim” and “weight” algorithms to test for GO‐term overrepresentation within the three domains: Biological process (BP), molecular function (MF) and cellular component (CC). The three algorithms differ in that the “classic” algorithm processes each GO‐term independently without considering the GO‐graph, the “elim” algorithm processes the GO‐graph bottom‐up while discarding genes that have already been mapped to significant GO‐terms, and the “weight” algorithm is weighing genes annotated to a GO‐term based on the scores of neighboring GO‐terms (Alexa et al., 2006). Based on simulated data, the “weight” algorithm has been shown to produce less false positives than the “classic” algorithm, whereas the “elim” algorithm further reduces false‐positive rate, but with a higher risk of discarding true positives (Privman et al., 2012). The *D. nivalis* annotated gene set was used as a custom background for all GO term enrichment tests. The significance level was set to *p* < .05, and the results were not corrected for multiple testing following the recommendations of the creators of the topGO package (Gentleman et al., 2004).

## 3 RESULTS

### 3.1 Genome assembly

**FIGURE 1:**
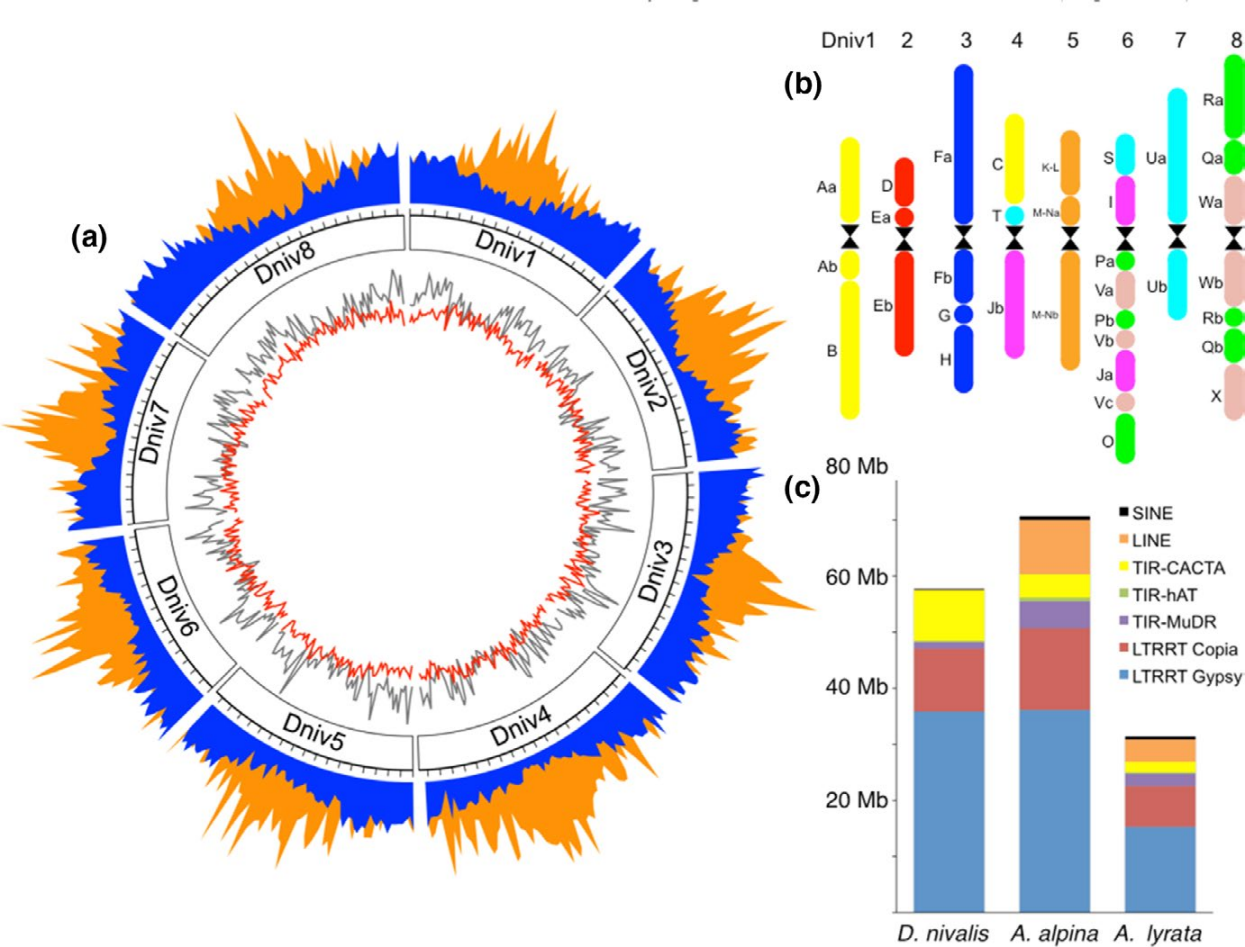
The*D. nivalis*genome assembly. (a) Circos plot of the eight chromosomes (Dniv1–Dniv8) showing the distribution of gene annotations (blue) and LTR‐RT elements (orange) in 500 Kb windows. Ticks represent 2 Mb intervals. The inner tracks show the distribution of TIR (grey) and Helitron (red) elements in 500 Kb windows. (b) Organization of ancestral Brassicaceae genomic blocks in the eight*D. nivalis*chromosomes (Dniv1–8) based on CCP and comparative analyses relative to*A. lyrata*and*A. alpina*. Centromere positions of chromosomes 1–3 and 8 are tentative, but supported by results in Figures S2, S3and the structure of other Arabideae species (Mandáková et al., 2020). Genomic blocks are coloured to match eight colours corresponding to eight chromosomes of the Ancestral Crucifer Karyotype (Lysak et al., 2016). (c) Relative abundance of TE superfamilies in selected species (see Table S5; Willing et al., 2015). Whereas LTR‐RT abundance is similarly elevated in*D. nivalis*and*A. alpina*relative to*A. lyrata*, LINEs appear to be reduced and TIR‐CACTA elements enriched in*D. nivalis*relative to both species [Colour figure can be viewed at wileyonlinelibrary.com]

Based on the 25mer frequency distribution we estimated the genome size of *D. nivalis* to 278.48 Mb (Figure S4; flow cytometry estimates report 254–308 Mb; Grundt et al., 2005). The initial de novo draft assembly (based on the Illumina paired‐end HiSeq data) had a scaffold N50 of 30.083 Kb and a length of 280.94 Mb. Scaffolding this assembly with 1.57 Gb of Chicago proximity ligation data using the HiRise pipeline resulted in a scaffold N50 of 2.92 Mb and a length of 300.29 Mb. Scaffolding with 1.33 Gb of Oxford Nanopore MinION long read data (207,896 reads ranging in length from 1 Kb to 158.14 Kb with a mean read length of 3.8 Kb) further improved the scaffold N50 to 4.44 Mb and the length to 301.71 Mb (Table 1; Table S3). We also produced a linkage map using 480 F_2_ individuals genotyped with 5,055 SNPs (Figure S1) to order scaffolds into eight pseudomolecules, referred to as chromosomes (see Methods). The final assembly is 301.64 Mb (scaffold N50 = 31.02 Mb), of which 276.24 Mb is anchored to chromosomes varying from 29.2 Mb to 43.1 Mb (Figure 1a).

**TABLE 1:** Assembly statistics for the *D. nivalis* genome. Each row shows a different stage in the scaffolding process of the genome assembly. For a more complete table, see Table S3

Assembly	Number of scaffolds (>1 Kb)	N50 (Kb)	L50	Longest scaffold (Kb)	Length (Including Ns; Mb)	Number of Ns per 100 Kb
DDN	28,339	30	2,663	203	281	51.58
DDN_Chicago	7,857	2,918	30	9,273	300	6,486
DDN_Chicago_ONT	6,765	4,437	21	20,017	302	6,874
DDN_Chicago_ONT_map	6,682	31,019	5	43,070	302	6,853

### 3.2 Chromosome evolution

**FIGURE 2:**
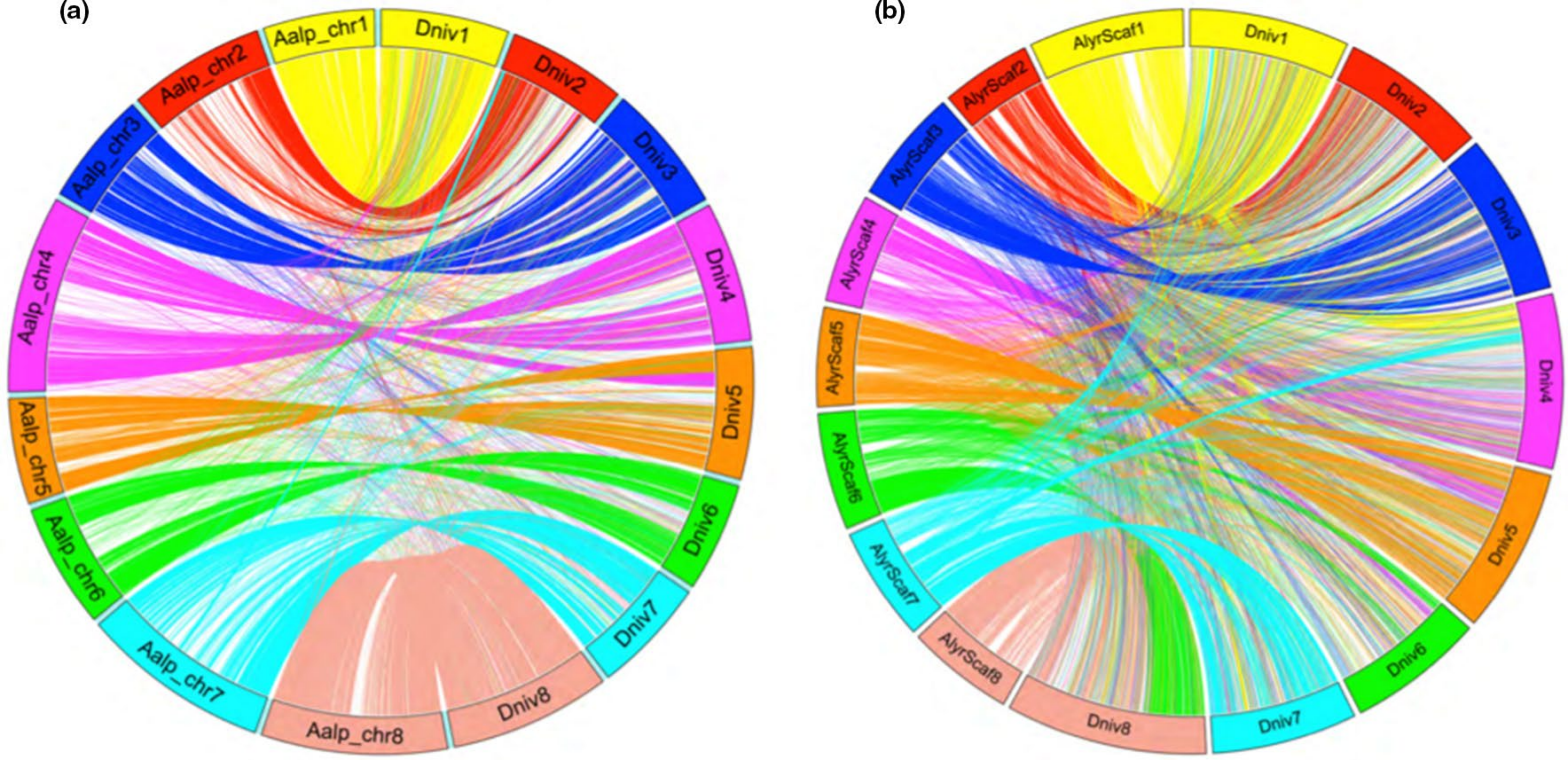
Syntenic relationships between*D. nivalis*and*Arabis alpina*(a) and*Arabidopsis lyrata*(b). The*D. nivalis*genome was aligned to the genomes of*A. alpina*and*A. lyrata*with NUCmer. Chromosomes are colour‐coded to match the Ancestral Crucifer Karyotype (ACK; Lysak et al., 2016) structurally resembling the*A. lyrata*genome (b) [Colour figure can be viewed at wileyonlinelibrary.com]

**FIGURE 3:**
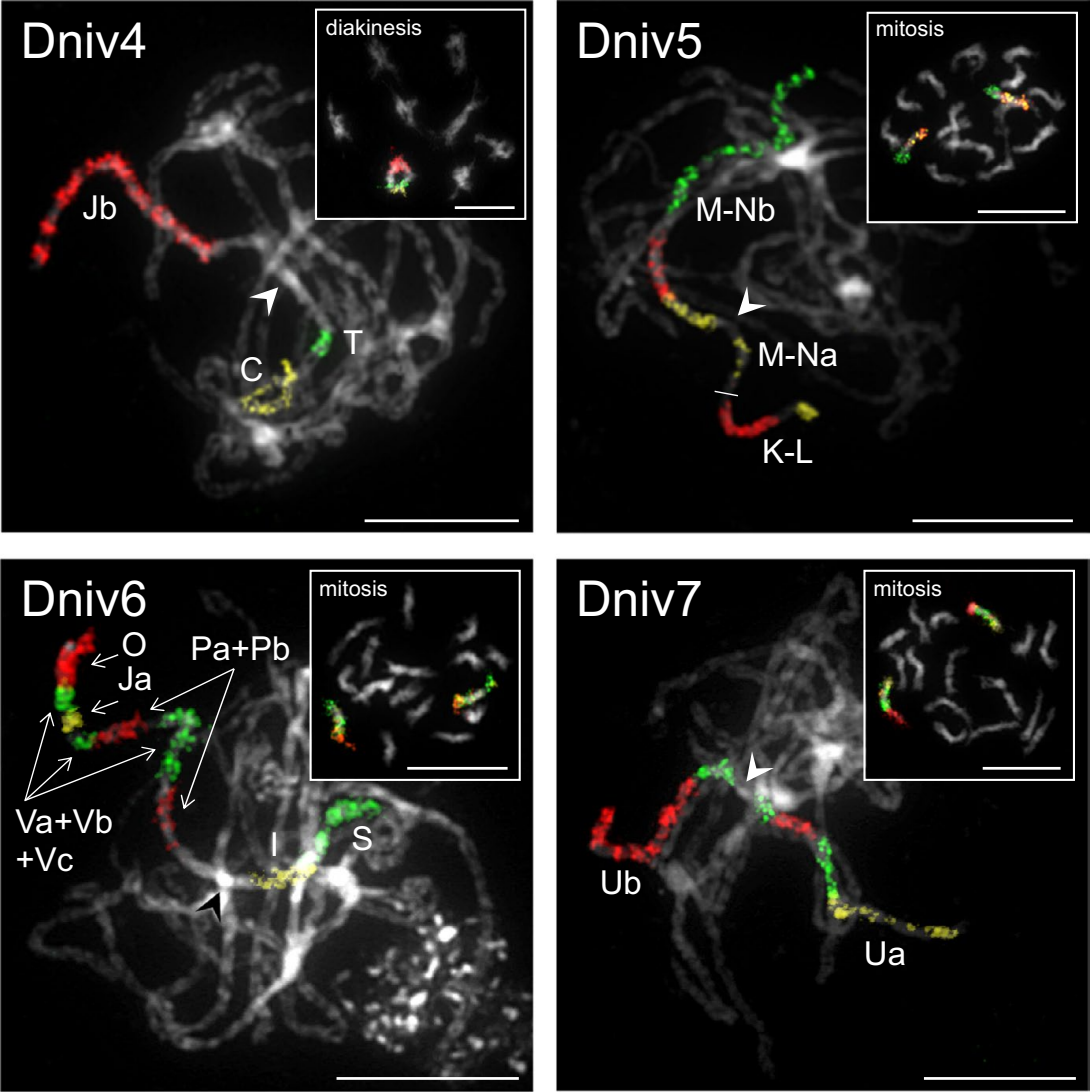
Detailed CCP analysis of*D. nivalis*chromosomes Dniv4, Dniv5, Dniv6, and Dniv7.*Arabidopsis thaliana*BAC contigs were in situ hybridized to pachytene chromosome spreads in*D*.*nivalis*(insets show the same probes localized on diakinetic chromosomes or mitotic metaphase chromosomes). Arrowheads indicate the position of centromeres. Green, yellow, and red colour corresponds to fluorescence of Alexa 488, Cy3 and Texas Red, respectively. Chromosomes were counterstained with DAPI. Scale bars 10 μm

To examine how the *D. nivalis* genome conforms to broader patterns of genome evolution in the Brassicaceae, we compared pairwise synteny between chromosomes of *D. nivalis* and those of *Arabidopsis lyrata* and *Arabis alpina*, and performed comparative chromosome painting (CCP) experiments to identify genomic blocks of the Ancestral Crucifer Karyotype (ACK, Schranz et al., 2006, Lysak et al., 2016; represented by the *A. lyrata* genome; Figures 1b, 2, and 3; Figures S2 and S3). By synthesizing these results, we inferred the structure of the *D. nivalis* chromosomes. We identified several rearrangements and extensive centromere repositioning relative to the ACK. The structure of the *D. nivalis* genome is very similar to that of *A. alpina* (Willing et al., 2015), the closest relative of *D. nivalis* for which a chromosome‐scale genome assembly is available, and consistent with genome structures determined for other Arabideae species, including three *Draba* species (Mandáková et al., 2020).

### 3.3 Repetitive element annotation

**FIGURE 4:**
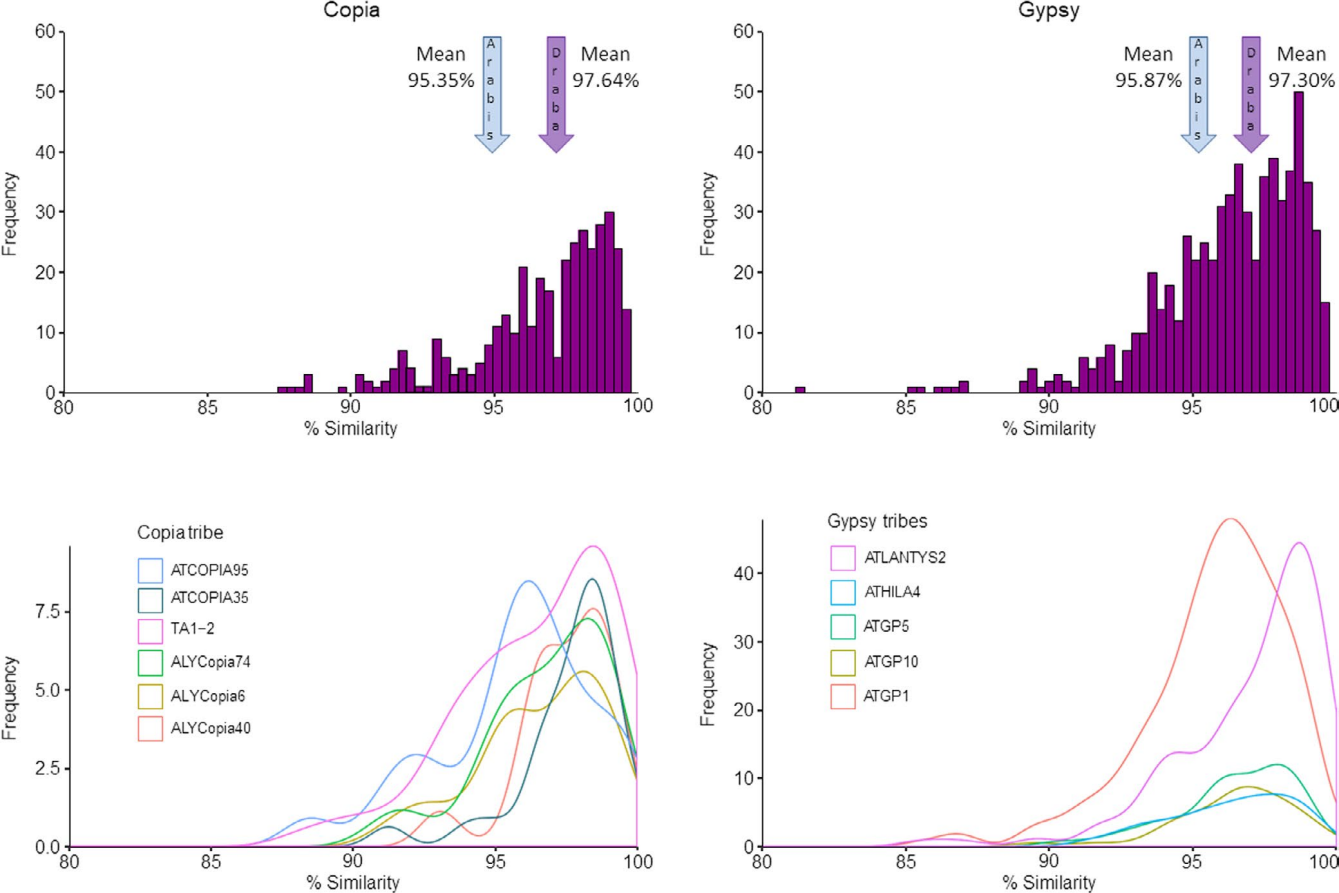
Evolutionary dynamics of abundant LTR‐RT tribes in*D. nivalis*. Results are based on the percent nucleotide similarity of LTR sequences among full‐length copies in the genome. Selected tribes show peaks indicative of transposition bursts that were above the average of all tribes for*Copia*and*Gypsy*(97.64% and 97.30%, respectively), distinguishing retrotransposons such as ATCOPIA95 showing ancestral proliferation from tribes such as ATCOPIA35 with a majority of recent transposition events. Summary statistics of all LTR‐RT tribes in*D. nivalis*and*A. alpina*are provided in Table S6 [Colour figure can be viewed at wileyonlinelibrary.com]

We annotated 94.8 Mb of the genome as direct remnants of repetitive elements, dominated by long terminal repeat retrotransposons (LTR‐RT, 60.1 Mb), terminal inverted repeat transposons (TIR, 20.9 Mb), and Helitrons (13.4 Mb; Figure 1; Tables S4–S6). Consistent with *A. alpina*, *A. lyrata*, and *A. thaliana*, LTR‐RT density increases in pericentromeric regions of each chromosome, TIR density decreases in pericentromeric regions, and Helitron density is stable along chromosomes. Abundance of LTR‐RT *Copia* and *Gypsy* elements is similar to that of *A. alpina* (Choudhury et al., 2017), whereas TIR‐CACTA elements and Helitrons seem to be particularly abundant in *D. nivalis* (Hu et al., 2019). Nucleotide divergence among LTR‐RTs identifies several *Copia* and *Gypsy* LTR‐RTs showing recent transposition bursts across the genome of *D. nivalis*. Some abundant LTR‐RTs (e.g., ALYCopia74, ATLANTYS2) have very similar copies (most > 98%) and thus seem to have proliferated more recently than in *A. alpina* (Figure 4). These results show that *Copia* elements, including the heat‐activated ATCOPIA78 (Ito et al., 2011) and tribes preferentially transposing across the gene space of Brassicaceae (Quadrana et al., 2016), have specifically contributed to the evolution of the *D. nivalis* genome.

### 3.4 Gene annotation

We predicted gene models with the Maker2 pipeline using BLAST homology to five Brassicaceae genomes and a de novo transcriptome assembly of *D. nivalis* based on RNA‐seq data from leaves, roots, flowers, and flower buds (see Methods). We identified 33,557 gene models, and 74% of the genes were functionally annotated based on similarity to UniProtKB entries, and 70% were annotated with InterPro domains. Approximately 58% of the 33,557 gene models had an annotation edit distance less than or equal to 0.25, suggesting a relatively high degree of agreement between predicted gene models and external evidence. This gene set is somewhat larger than that of *A. thaliana* (27,654), but consistent with those of closely related species with similar genome size (Figure S5), and BUSCO analyses indicate 95.2% completeness of conserved embryophyte genes (Table S7). The average gene density in *D. nivalis* is approximately one gene per 9 Kb, and similar to *A. thaliana*, *A. lyrata*, and *A. alpina*, gene density decreases towards the centromeres (Figure 1a; Willing et al., 2015).

### 3.5 Gene and gene family evolution

**FIGURE 5:**
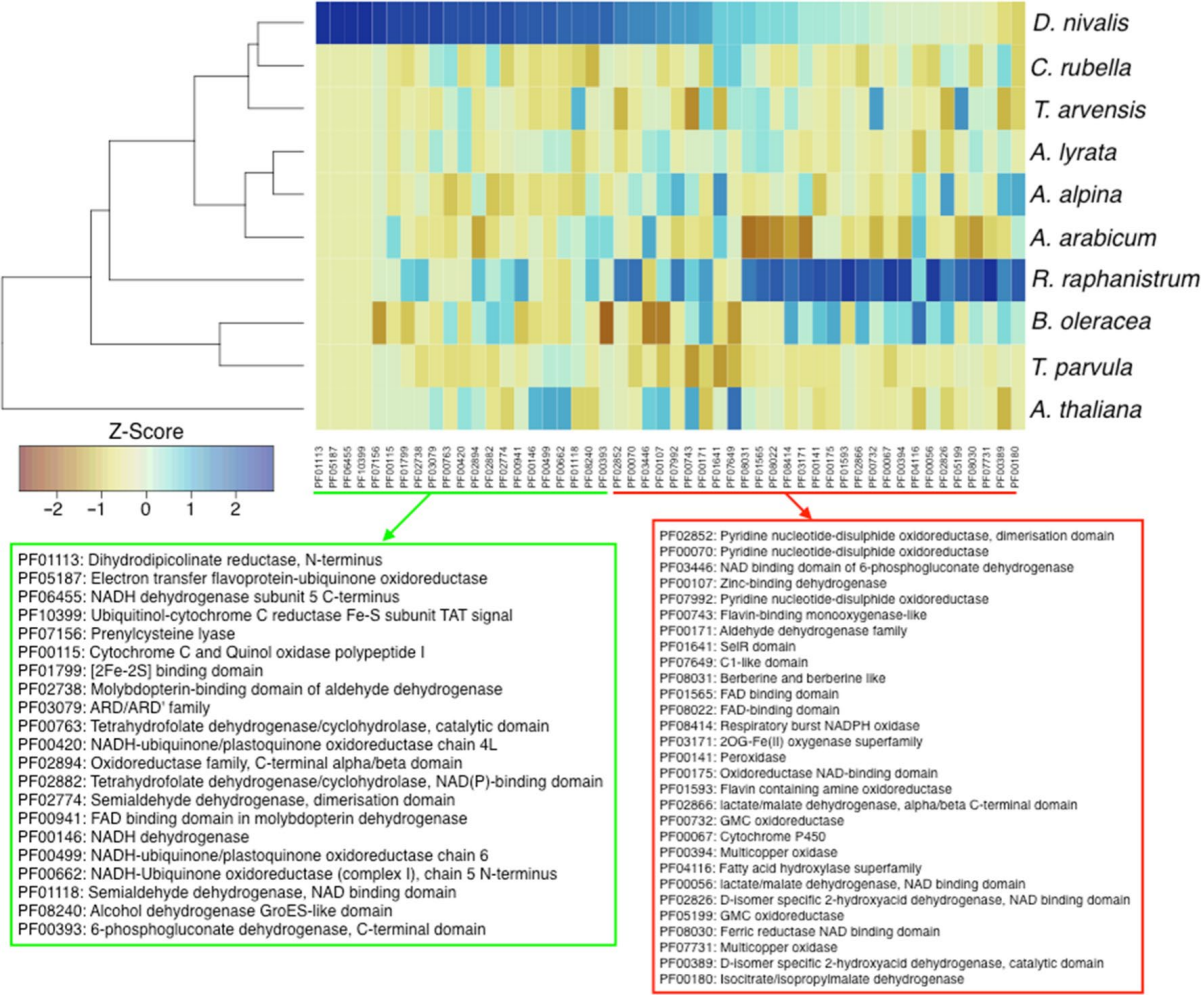
Pfam domains associated with oxidation‐reduction processes are enriched in*D. nivalis*. Heatmap comparing Pfam domains annotated with the BP GO term “oxidation‐reduction process” (GO:0055114) in selected Brassicaceae species. Cells are coloured by Z‐score. The dendrogram on the left represents groupings based on similar counts of selected Pfam domains. Pfam domains detailed in green are significantly enriched in*D. nivalis*relative to the other nine species (Z‐score > 1.96). Pfam domains detailed in red are not significantly enriched, but have > 10 genes annotated in*D. nivalis*. The genomes of both*D. nivalis*and*R. raphinistrum*contain relatively abundant and significantly enriched Pfam domains associated with oxidation‐reduction processes, which are important in stress response signalling pathways [Colour figure can be viewed at wileyonlinelibrary.com]

To explore specialization in the *D. nivalis* gene set, we compared the abundance of protein family (Pfam) annotations with those of nine Brassicaceae genomes representing broad phylogenetic sampling. We found 226 Pfam domains to be significantly enriched and 32 to be significantly depleted relative to the other species (Table S8). To summarize functional associations of the enriched Pfam domains, we extracted gene ontology (GO) terms from their corresponding InterPro entries. Amongst these GO terms, the most common biological process (BP) GO term is “oxidation‐reduction process”. Numerous environmental stimuli and stresses can lead to the production of reactive oxygen species (ROS), which can damage cell membranes, nucleic acids, proteins, and metabolites (Apel & Hirt, 2004). Regulation of ROS metabolism is essential for maintaining cellular oxidation‐reduction (redox) homeostasis and is an integral part of the intracellular signal transduction networks evoked by external stimuli (Mittler, 2017), particularly for responses to environmental stresses induced by light, drought, and cold (Neill et al., 2002). The significant increase in Pfam annotations involved in redox processes in *D. nivalis* may indicate that it has evolved novel ways to cope with ROS accumulation associated with Arctic environmental stress (Figure 5). The salt tolerant *Eutrema salsugineum* (syn. *Thellungiella salsuginea*), for example, responds to salt stress by expressing an aldehyde dehydrogenase, a scavenger of toxic aldehydes produced as a byproduct of ROS accumulation (Hou & Bartels, 2014). Consistent with this, the *D. nivalis* annotated gene set contains 26 genes containing the significantly enriched molybdopterin‐binding domain of aldehyde dehydrogenase.

**FIGURE 6:**
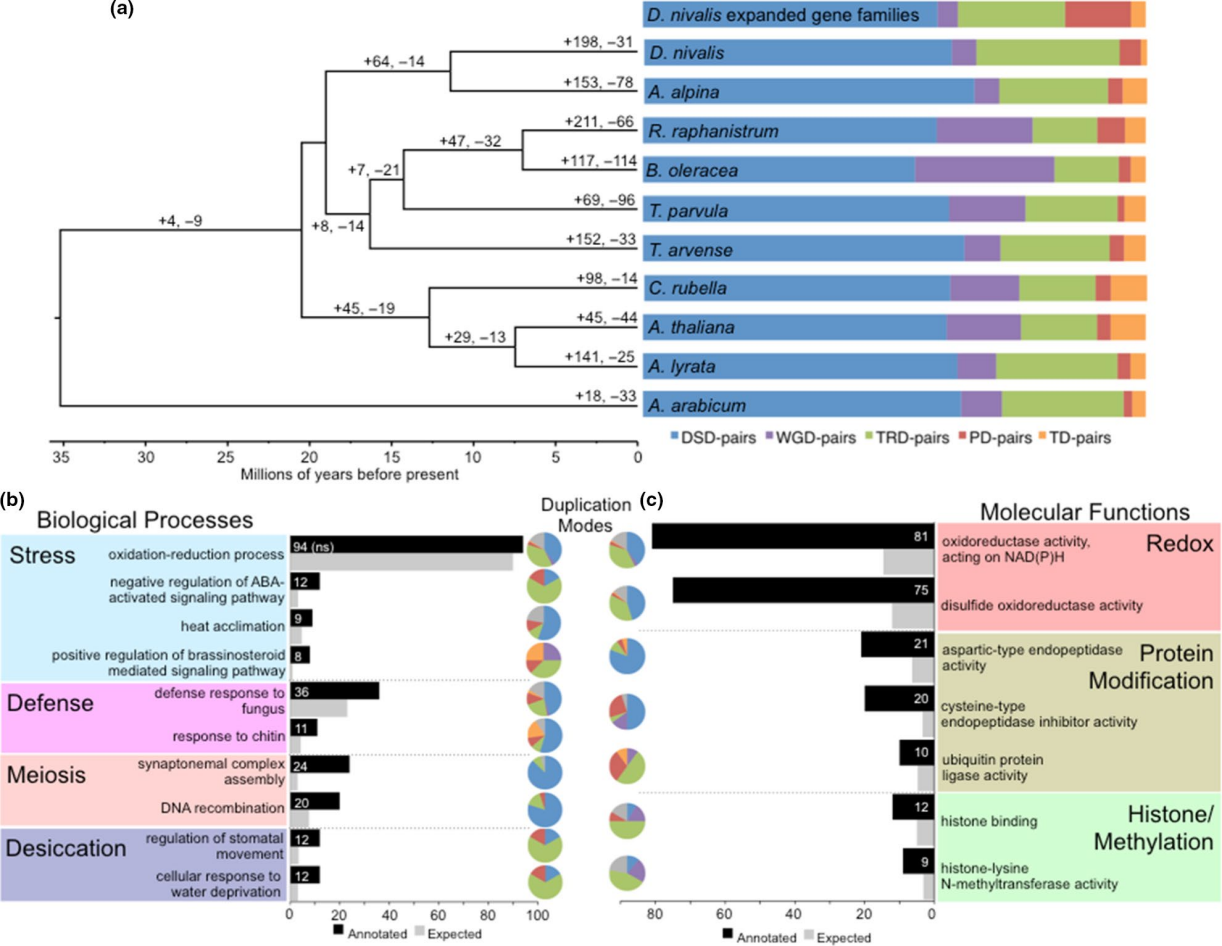
Gene family evolution in*D. nivalis*. (a) Time‐calibrated species tree of 10 Brassicaceae genomes showing branches labelled with significant gene family expansions and contractions (+gene families gained, ‐gene families lost). To the right we show the percentage of gene pairs derived from different modes of duplication in the 10 species. For*D. nivalis,*gene duplication modes are shown for both the whole genome (labelled*D. nivalis*) and for the 2,645 genes that constitute 198 significantly expanded gene families (EGFs). Patterns of gene duplication are broadly consistent between the whole genome and the EGFs, except that transposed (TRD) duplications are less frequent (*p*‐value = 1.8e‐15), and proximal (PD,*p*‐value < 2.2e‐16) and tandem (TD,*p*‐value = 2.432e‐10) duplications are more frequent in EGFs than would be expected by chance (Fisher's exact test). Values for all species other than*D. nivalis*are from Qiao et al. (2019), and since their analysis did not include*R. raphanistrum*, the results shown are from the closely related*Raphanus sativa*. DSD, dispersed duplication; WGD, whole‐genome duplication; TRD, transposed duplication; PD, proximal duplication; TD, tandem duplication. (b and c**)**Select biological process (b) and molecular function (c) GO terms significantly enriched (Fisher's exact test*p*‐value < 0.05) in*D. nivalis*EFGs (in b, oxidation‐reduction process was not significant, indicated by “ns”). Terms are grouped into broad categories to simplify interpretation. Pie charts for each term show the modes of gene duplication inferred for genes annotated with these terms (DSD, WGD, TRD, PD, TD; colour scheme follows a, with unclassified in grey) [Colour figure can be viewed at wileyonlinelibrary.com]

To compare the diversity and abundance of *D. nivalis* gene families relative to the nine other Brassicaceae species, we estimated gene family (orthogroup) membership using OrthoFinder. A total of 29,194 (87%) *D. nivalis* genes were assignable to one of the 21,635 gene families identified, and 10,401 of the gene families contain at least one gene copy in all 10 species. Genome‐wide classification of gene duplications in *D. nivalis* using the Dup_GenFinder pipeline (Qiao et al., 2019) resulted in similar patterns across all species (Table S9). Gene duplications in *D. nivalis* are dominated (22,989 gene pairs, 89.6%) by transposed (TRD, 7,308 pairs) and dispersed (DSD, 15,681 pairs) duplicates (Figure 6a), both of which are probably the product of transposition events that can be mediated by transposable elements (Qiao et al., 2019).

Relative to the nine other species, *D. nivalis* contains 198 significantly expanded and 31 significantly contracted gene families (Figure 6a). Exploring the functional annotations of the 2,958 genes of the expanded gene families (EGFs), we found 158 significantly enriched BP GO annotations including several functions highlighting how this species was able to adapt to Arctic habitats (Figure 6b; Tables S10–S12). Functions associated with stress signaling include both the abscisic acid (ABA) activated and brassinosteroid‐mediated pathways, involved in cellular functions including abiotic stress signaling (Planas‐Riverola et al., 2019). We also see functional enrichment for heat acclimation associated with three EGFs, which also are associated with defense responses to fungal pathogens. While fungal pathogens are not expected to be particularly virulent in the Arctic, stress can make plants more susceptible to pathogens. The EGFs are also enriched for functions associated with meiosis, specifically the assembly of the synaptonemal complex. The efficiency and fidelity of recombination is sensitive to temperature (Bomblies et al., 2015), and these results may indicate adaptation in *D. nivalis* to facilitate meiosis in cold habitats. Gene families associated with desiccation resistance are also expanded in *D. nivalis*, consistent with its occurrence in extremely dry, so‐called polar deserts. While BP terms related to redox homeostasis were not enriched in the EGFs (Figure 6b), redox activity was prominent among the 64 enriched MF terms (Figure 6c), consistent with the overrepresentation of redox Pfam domains observed in the genome (Figure 5). Functions associated with protein modification and ubiquitination were enriched in the EGFs, consistent with previously published results for the salt tolerant *Thellungiella salsuginea* (Yang et al., 2013). Finally, the *D. nivalis* genome contains several EGFs that function in histone binding and methylation, integral parts of epigenetic regulatory mechanisms that can play important roles in numerous abiotic stress signaling and response pathways (Ueda & Seki, 2020). Patterns of duplication inferred for the 2,958 genes that constitute the EGFs in *D. nivalis* are broadly consistent with genomic patterns in that TRD and DSD duplications dominate (79.9%), but TRD duplications are less frequent, and proximal (PD) and tandem (TD) duplications are more frequent in EGFs than would be expected by chance (Figure 6a, Table S9). This suggests that the activity of prevalent LTR‐RTs, TIR transposons, and Helitrons probably played important roles in the expansion of *D. nivalis* gene families, but processes of proximal and tandem duplication also appear to have been important in the expansion of gene families associated with protein modification, stress signalling, desiccation resistance, and defense responses to fungal pathogens (Figure 6b,c).

### 3.6 Tests of positive selection

**FIGURE 7:**
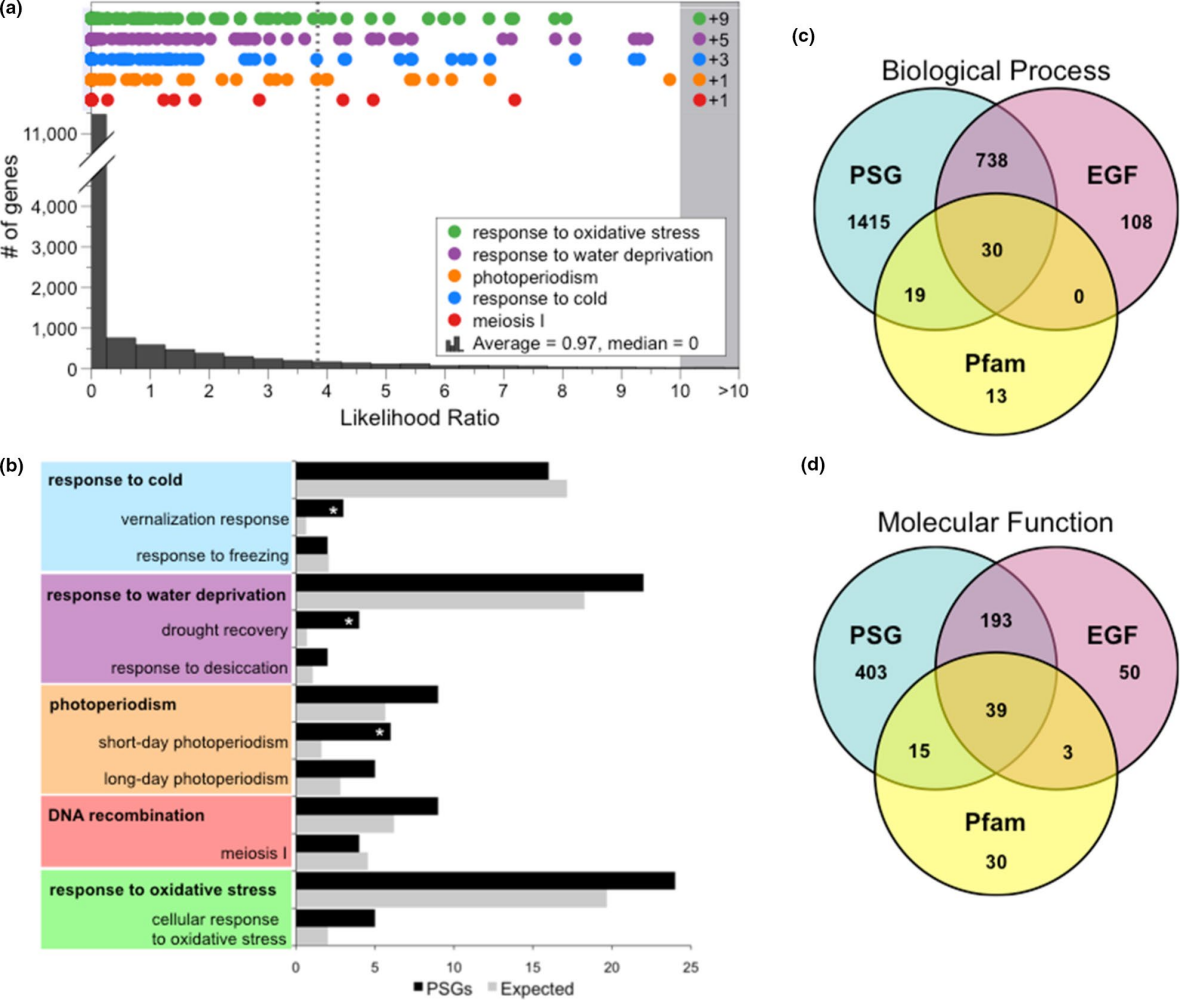
Genes under positive selection in*D. nivalis*. (a) Distribution of the ratios of ln likelihoods (lnL) from tests for positive selection in 15,828*D. nivalis*genes. Genes with a higher proportion of nonsynonymous to synonymous substitutions have a higher lnL ratio, and those with an lnL ratio above the*X* ^2^critical value (3.84, dashed line,*p*‐value < 0.05,*df*=1) are considered significantly likely to contain codons that evolved under positive selection in*D. nivalis*(PSGs; see Methods). Coloured dots represent genes that are annotated with biological process (BP) GO terms of particular interest for Arctic adaptation. (b) Summary of key BP GO terms in the*D. nivalis*PSGs. Asterisks (*) indicate significantly enriched terms relative to the genomic background. Parent terms are in bold. (c and d) Venn diagrams showing the overlap of BP (c) and molecular function (d) GO terms resulting from analysis of Pfam domains, expanded gene families (EGF), and PSGs in*D. nivalis*(see also Table S10) [Colour figure can be viewed at wileyonlinelibrary.com]

To search for further evidence of Arctic adaptation in *D. nivalis*, we performed genome‐wide positive selection tests to identify genes that probably evolved under positive selection in this lineage relative to seven related species (see Methods). We found 1,307 positively selected genes (PSGs). These include several candidate genes with functions directly relevant to typical environmental stresses of the Arctic, associated with “response to cold”, “response to water deprivation”, “photoperiodism”, “response to oxidative stress”, and “meiosis I” (Figure 7a,b; Tables S13 and S14). Patterns of functional enrichment of the PSGs also highlight several significant BP GO terms probably connected to Arctic adaptation, including “vernalization response”, “drought recovery”, “short‐day photoperiodism”, and “oxidation‐reduction process” (Figure 7b; Table S14). We also found four PSGs associated with meiosis I, including two *D. nivalis* homologues to *A. thaliana ZYP1A*, which is one of three synaptonemal complex transverse filament proteins whose function is disrupted by temperature stress (Bomblies et al., 2015). These results provide evidence for the likely adaptive evolution of core meiosis genes reflected both in EGFs (Figure 6b) and in positive selection acting on specific components of the synaptonemal complex.

## 4 DISCUSSION

Summarizing the results of our comparative genomic analyses, we observe some similarities in functional patterns among enriched Pfam domains, gene family expansions, and genes under positive selection (Figure 7c,d). Our results reveal a multifaceted landscape of stress adaptation in the *D. nivalis* genome, and highlight the important roles that genes involved in stress signaling/response, redox homeostasis, light sensing, and meiosis probably play in plant adaptation to the extreme Arctic environment. The numerous genes that we have identified represent good candidates for future studies of functional validation in various stress responses. If such studies are successful, they could provide guidance for various approaches to crop improvement. The highly contiguous genome assembly of *D. nivalis* that we have produced provides numerous avenues for the continued development of this species as the first Arctic specialist model plant. Future uses of this resource could include, e.g., studies of the adaptive potential of Arctic species to future climate change.

## AUTHOR CONTRIBUTIONS

A.K.B., C.B., M.D.N., L.R. conceived and initiated the study. M.D.N. performed the assembly and annotation of the *D. nivalis* genome. C.P., R.R.C. carried out the repeat analysis. M.A.L., T.M., X.G. conducted the CCP experiments. A.G., A.L.S.G., M.D.N., S.B. carried out experimental plant work for genome sequencing. A.G., A.S.N., A.L.S.G., M.D.N., S.B. produced and genotyped the F_2_ mapping population. A.L.S.G., M.F., M.D.N., T.S. constructed the genetic map. S.B. conducted the analyses of molecular evolution. M.D.N., S.B. carried out the comparative genomics analyses. M.D.N. wrote the manuscript with comments from all coauthors. A.K.B., C.B., L.R., M.D.N., T.S., supervised the study.

## Supporting information

Supplementary Material

Table S1–14

## Data Availability

The raw data (shotgun sequence data, MinION long reads, Chicago Linked Reads, and RNA‐seq) have been deposited in the NCBI SRA with Bioproject number PRJNA657155. The final chromosome‐scale assembly, gene annotation, repeat library and annotation, transcriptome assembly, ddRAD sequence data of the F2 mapping population, and vcf file of variants used in the construction of the genetic map are available on Dryad (https://doi.org/10.5061/dryad.pg4f4qrm4).
