# Supplementary material for: The genome of Draba nivalis shows signatures of adaptation to the extreme environmental stresses of the Arctic
Source: Mol Ecol Resour. 2020 Nov 12;21(3):661–76. doi: 10.1111/1755-0998.13280 (PMC7983928; doi:10.1111/1755-0998.13280)
Supplement: Supplementary file 1 — Supplementary Material [file MEN-21-661-s002.pdf]

Supplementary Figures

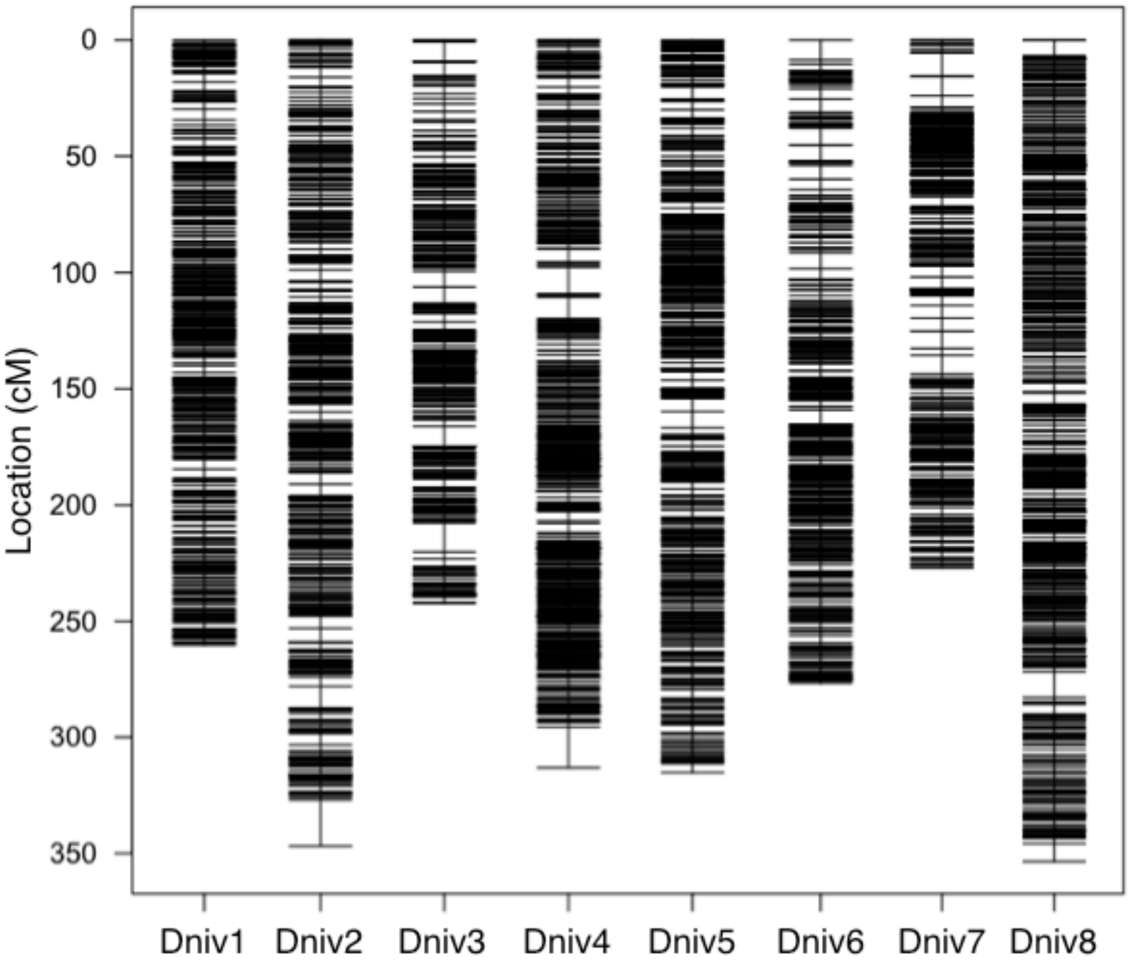

Supplementary Figure 1. Genetic linkage map of *D. nivalis*. Based on genotyping with 5,055 biallelic SNPs in 480 F_2 progeny resulting from self-pollination of an F_1 hybrid between *D. nivalis* accession 045-5 from Norway (maternal parent) and *D. nivalis* accession 008-7 from Alaska (paternal parent; this is the plant from which the reference genome assembly was produced; see methods). Numbering of chromosomes follows the final *D. nivalis* genome assembly (Figure 1). Map locations are provided in Supplementary Table 2.

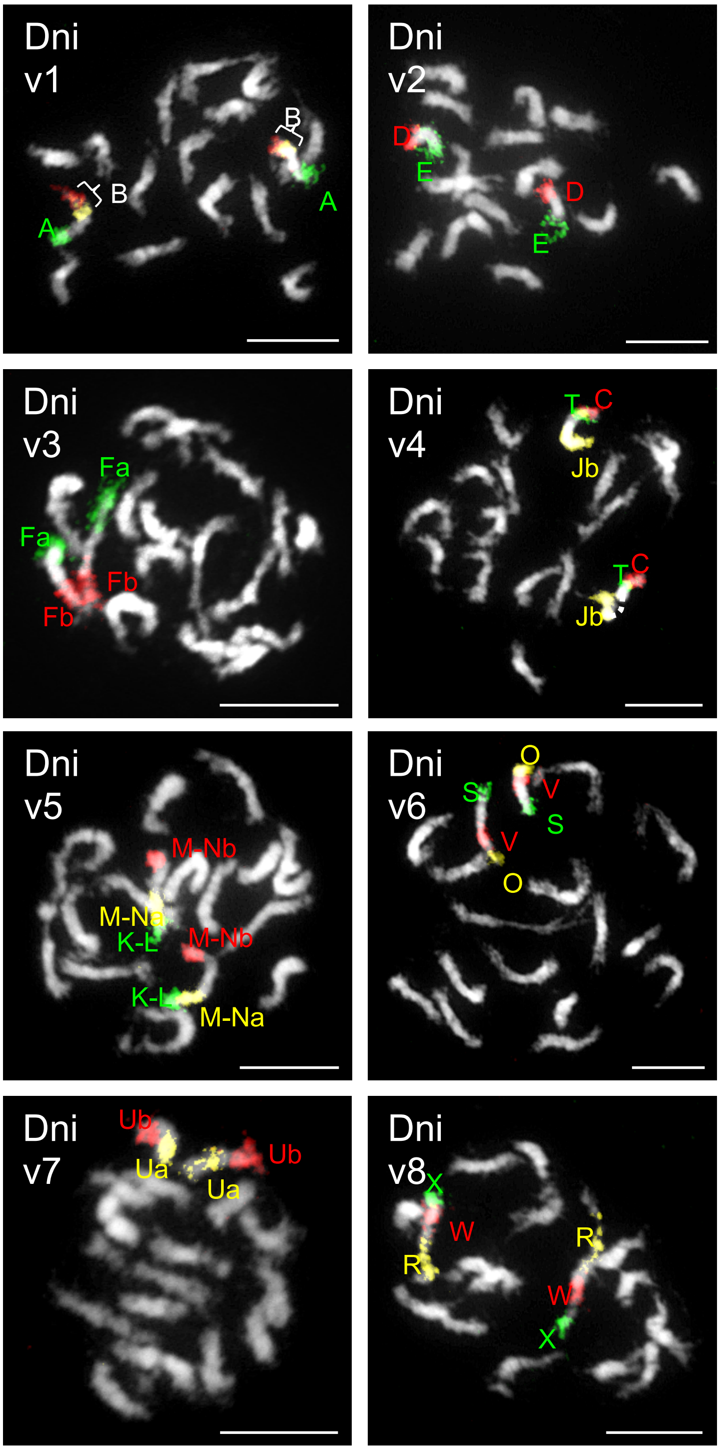

Supplementary Figure 2. Comparative chromosome painting (CCP) in *D. nivalis*. The structure of the eight *D. nivalis* chromosomes (Dniv1-Dniv8) reconstructed by *in situ* hybridization of probes for conserved Brassicaceae genomic blocks to mitotic prometaphase/metaphase chromosomes. Chromosomes were counterstained with DAPI. Scale bars 10 µm.

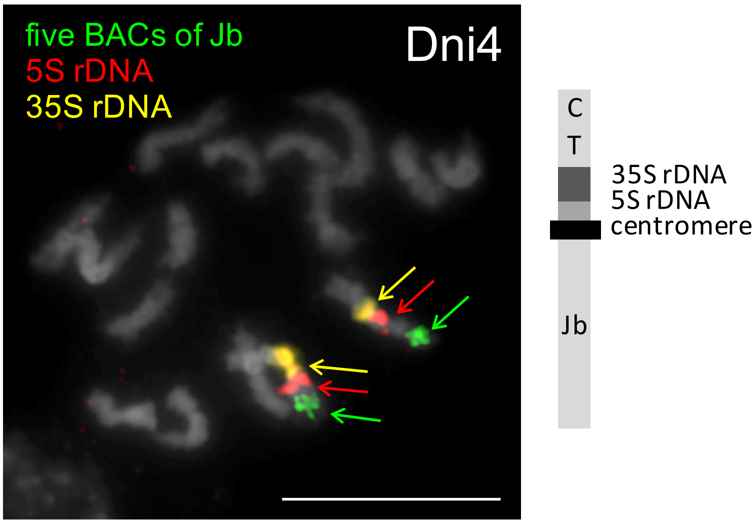

Supplementary Figure 3. Detailed CCP analysis of *D. nivalis* chromosome Dniv4. Localization of rDNA loci is shown on mitotic chromosomes. FISH probes for 5S rDNA, 35S rDNA and five *A. thaliana* BAC clones corresponding to the genome block Jb revealed both rDNA loci adjacently positioned on the upper arm of Dniv4. A graphical representation of the structure of Dniv4 is shown to the right. Chromosomes were counterstained with DAPI. Scale bar: 10 µm.

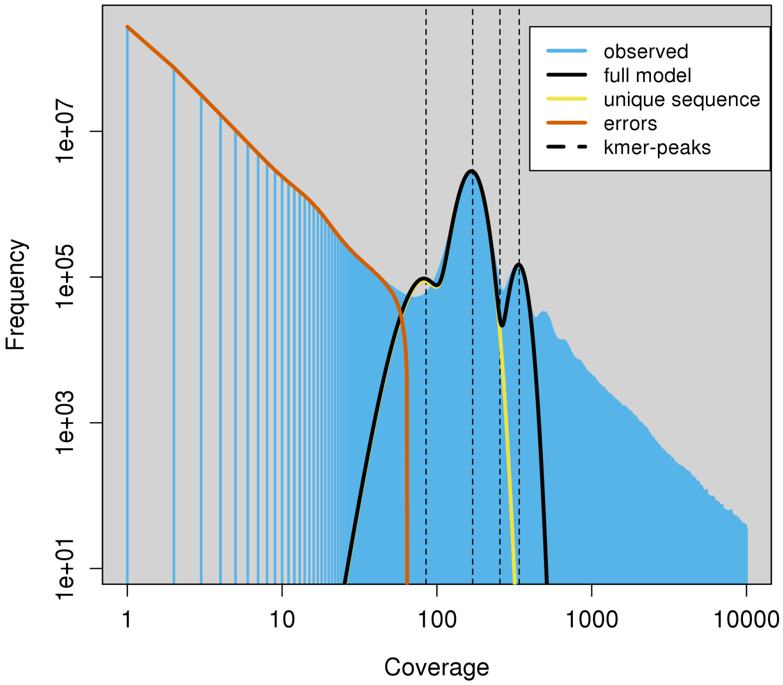

Supplementary Figure 4. Distribution of 25mer frequencies from approximately 134 million reads of 250 bp length from the whole genome shotgun sequence dataset. A genome size of 278.484 Mb was estimated. Figure produced using GenomeScope (Vurture *et al.* 2017; http://qb.cshl.edu/genomescope/).

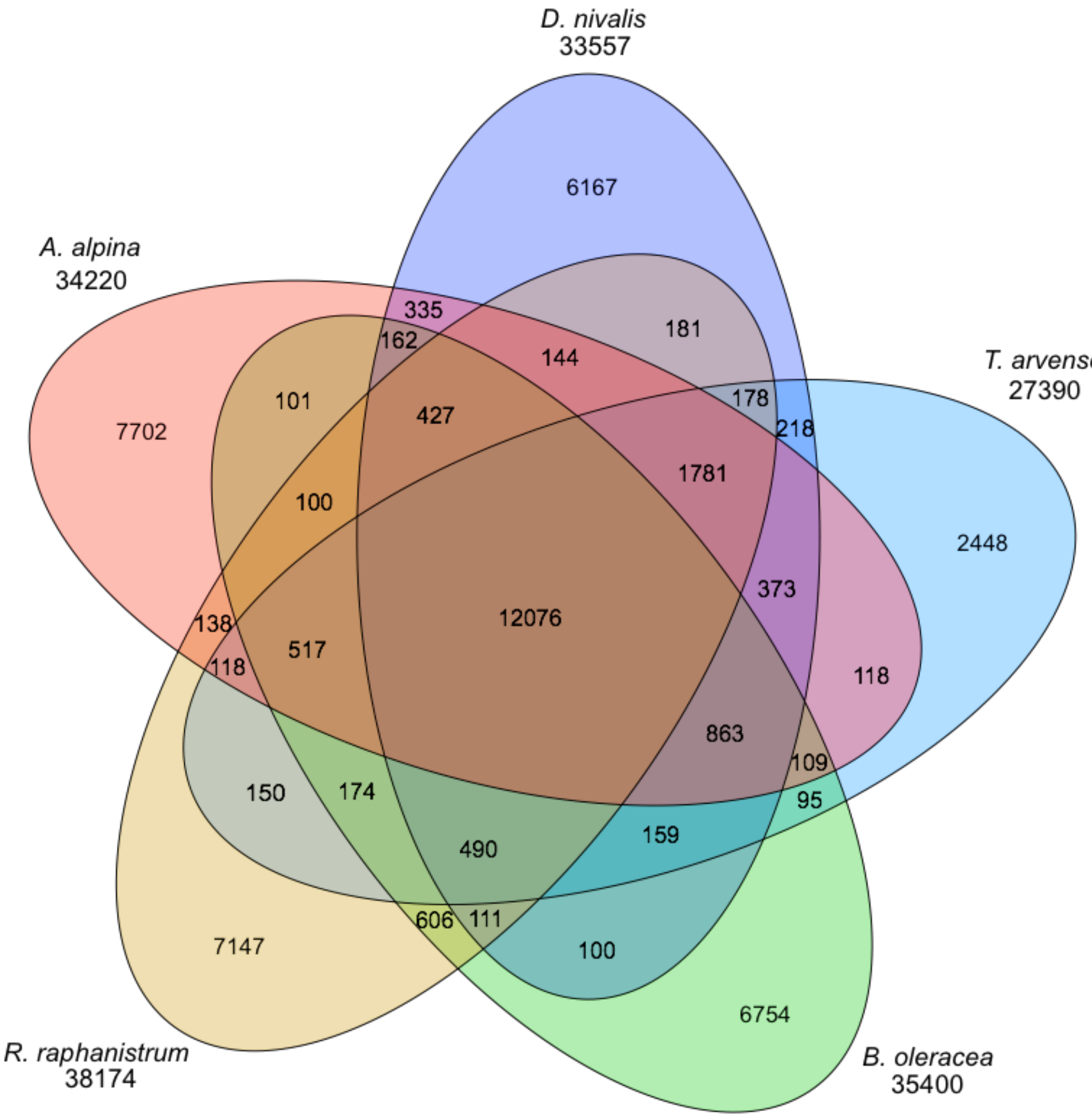

Supplementary Figure 5. **Venn diagram comparing gene family membership in Brassicaceae Clade B species.** Following the clade definition of Guo et al. (2017). The total annotated gene count for each species is shown below the species name.

Supplementary Methods

Protocol for ddRAD genotyping of F_2 mapping population. The digestion step was performed in 40 µl reactions containing 500 ng DNA, 1X CutSmart buffer, 10 U NsiI-HF (New England Biolabs [NEB], R3127) and 5 U MseI (NEB, R0525) in a thermal cycler at 37°C for 2 hrs. The DNA was cleaned using 1.2 volumes Ampure XP (Beckman Coulter) and eluted in 25 µl H_2O. Adapter ligation was performed in 30 µl reactions containing 1x T4 DNA ligase buffer (NEB), 400 U T4 DNA ligase (NEB, M0202), 2.66 µM indexed P1 and P2 adapter mix, and 24 µl digested DNA in a thermal cycler set to 25°C - 30 min, 65°C - 10 min and 4°C - hold. The samples to be sequenced together on an Illumina HiSeq lane were pooled, cleaned using 1.2 volumes Ampure XP and eluted in 240 µl 10 mM Tris-HCl. The libraries were amplified in a PCR reaction with 1x Q5 HiFi Master Mix (NEB, M0492), 0.5 µM of each primer, template DNA and nuclease-free water. We did not exceed the recommended DNA input amount stated by NEB for the Q5 polymerase, which is 1 µg template DNA per 50 µl PCR reaction. PCR conditions were 98°C – 30 sec, (98°C – 10 sec, 60°C – 15 sec, 72°C – 15 sec) x 8, 72°C – 2 min. The libraries were cleaned using 0.8 volumes Ampure XP. The DNA had to be cleaned up to three times to remove all short fragments (<200 bp).

References

Guo, X. *et al.* Plastome phylogeny and early diversification of Brassicaceae. *BMC Genomics* **18,** 176 (2017).

Schranz, M. E., Lysak, M. A. & Mitchell-Olds, T. The ABC's of comparative genomics in the Brassicaceae: building blocks of crucifer genomes. *Trends in Plant Science* **11,** 535–542 (2006).

Vurture, G. W. *et al.* GenomeScope: fast reference-free genome profiling from short reads. *Bioinformatics* **33**, 2202–2204 (2017).
